# A novel animal model of osteonecrosis of the femoral head based on 3D printing technology

**DOI:** 10.1186/s13018-023-04050-7

**Published:** 2023-08-03

**Authors:** Yiyang Li, Jiewen Zhang, Yiwei Zhao, Run Tian, Pei Yang

**Affiliations:** Second Affiliated Hospital of Xi’an Jiaotong University, Xi’an, China

**Keywords:** Animal model, Osteonecrosis of the femoral head, Rabbit, 3D printing technology

## Abstract

**Background:**

Osteonecrosis of the femoral head (ONFH) is a prevalent orthopedic condition characterized by the disruption of blood supply to the femoral head, leading to ischemia of internal tissues, subchondral bone fractures, necrosis, and eventual collapse of the weight-bearing portion of the femoral head. This condition results in severe functional impairment, pain, and even disability of the hip joint. Existing animal models of ONFH have limitations in replicating the natural disease progression accurately. Thus, there is a critical need to develop a novel animal model capable of better simulating localized pressure on the human femoral head to facilitate ONFH-related research.

**Methods:**

In this study, we present a novel approach for modeling ONFH, which involves integrating stress factors into the modeling process through the utilization of 3D printing technology and principles of biomechanics. A total of 36 animals were randomly assigned to six groups, where they received either the novel modeling technique or the traditional hormone induction method. Subsequently, an 8-week treatment period was implemented, followed by conducting micro-CT scans and histological evaluations to assess tissue outcomes.

**Results:**

The study evaluated the cytotoxicity of the material used in the new model, and it was observed that the material did not exhibit any cytotoxic effects on cells. Additionally, the novel model successfully replicated the pathological features of ONFH, including femoral head collapse, along with a substantial presence of empty bone lacunae, cartilage defects, and subchondral bone fractures in the subchondral bone region.

**Conclusion:**

In conclusion, our study provides evidence that the new model shows the ability to simulate the progression of the disease, making it a valuable tool for research in this field and can contribute to the development of better treatment strategies for this debilitating condition. It holds great promise for advancing our understanding of the pathogenesis of ONFH and the potential therapeutic interventions for this challenging clinical problem.

## Introduction

Osteonecrosis of the femoral head (ONFH) is one of the most common orthopedic diseases in clinical practice [1, 2]. It is characterized by an interruption of blood supply to the femoral head due to venous stasis or arterial insufficiency, leading to ischemia of the internal tissues of the femoral head [3]. This, in turn, can result in subchondral bone fracture, necrosis, and collapse of the weight-bearing portion of the femoral head, causing severe functional impairment, pain, and even disability of the hip joint [4]. Non-traumatic avascular necrosis of the femoral head (NONFH) is the main cause of disability in young people, with the misuse of hormones and alcohol greatly increasing its incidence [5, 6]. Currently, femoral head collapse is considered to be the final stage of NONFH [7]. The severe pain and functional impairment caused by femoral head collapse greatly affect patients' quality of life, making the prevention and delay of femoral head collapse an effective approach to preventing and treating NONFH [8, 9].

Current research indicates that the construction of animal models for ONFH can be broadly categorized into several types of modeling methods, including physical methods [10], alcohol-induced methods [11], hormone-induced methods [12], and surgical trauma-induced methods [13]. Nevertheless, these models suffer from the drawback of inducing widespread femoral head necrosis, and to date, no animal model has been established to fully recapitulate the pathological and physiological processes of human ONFH. This represents a significant impediment to the advancement of femoral head necrosis research [14]. Current research suggests that the initiation of collapse in end-stage ONFH may be attributed to microfractures in trabeculae during bone remodeling, which lead to gradual changes in internal mechanical properties and consequent concentration of stress on the trabeculae. This results in the acceleration of bone absorption and establishes a vicious cycle [15]. Based on hip joint movement characteristics, the femoral head's stress column can be classified into the medial, superior, and lateral columns. The lateral column of the femoral head is believed to be a crucial predictive factor for the collapse of ONFH [16].

Currently, the rabbit ONFH model lacks specific investigations into local compression of the femoral head. A novel animal model for ONFH is needed based on a new biomechanical understanding. Therefore, this study aimed to establish a new animal model of ONFH that can simulate the local compression of the human femoral head, providing a fundamental basis for ONFH-related research.

## Material and methods

### Femoral head compression devices design, fabrication, and characterization

#### Design and fabrication

**Fig. 1:**
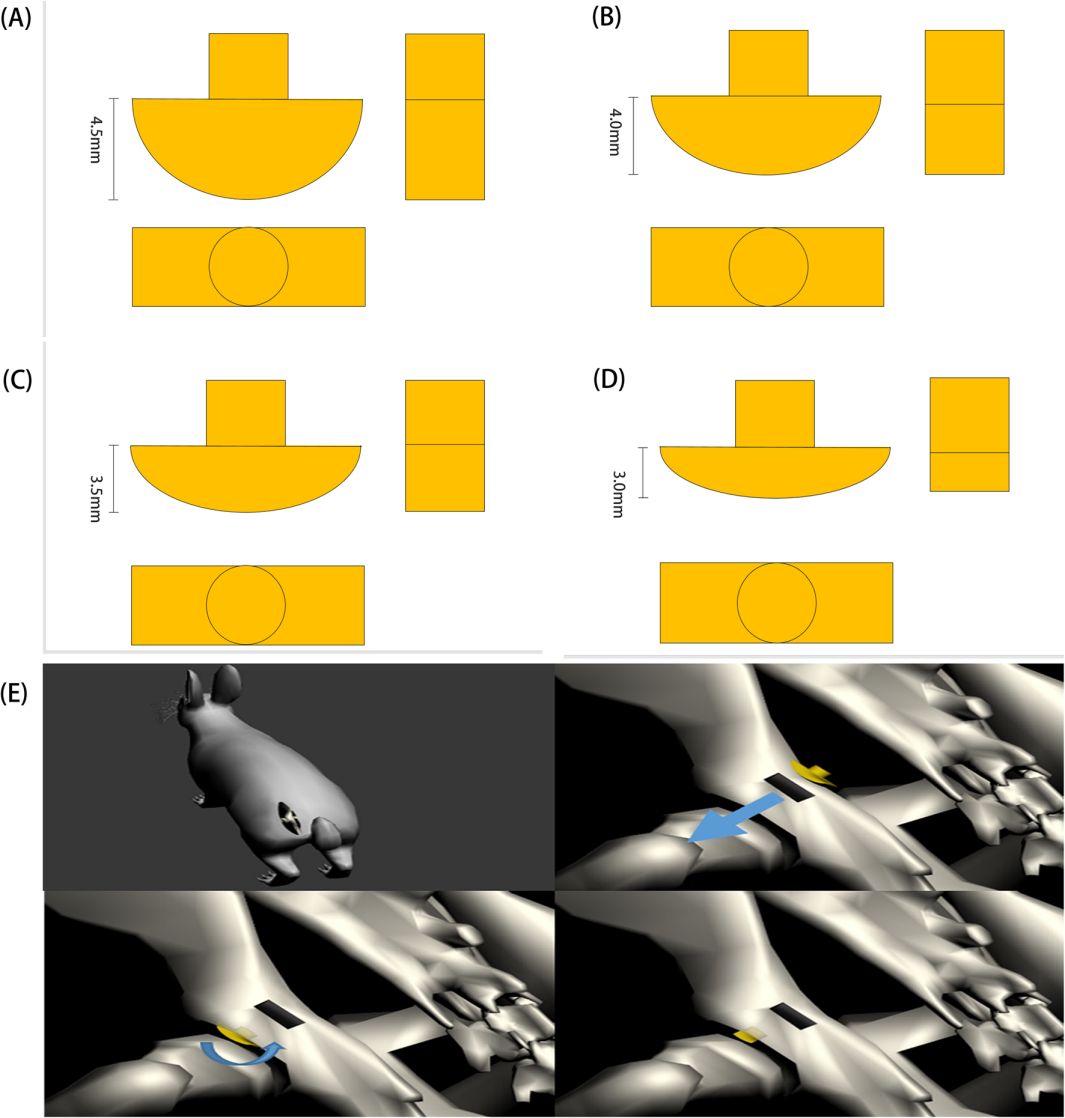
**A** 4.5 mm intraacetabular femoral compression nails, **B** 4.0 mm intraacetabular femoral compression nails, **C** 3.5 mm intraacetabular femoral compression nails, **D** 3.0 mm intraacetabular femoral compression nails. **E** Implantation fixation system for femoral head compression device

Utilizing a 3D printing process (ZRapid Tech. China) and resin as the principal material to manufacture a pressurization device. A compression device was developed with dimensions of a long axis of 6 mm and a short axis of 2 mm, informed by the rabbit acetabulum’s thickness and the gap within it. The thickness of the compression device was found to be positively correlated with the pressure it exerted on the rabbit femoral head. To explore this relationship, four compression devices of varying thicknesses (4.5 mm, 4.0 mm, 3.5 mm, and 3.0 mm) were designed and fabricated (Fig. 1A–D). To secure the compression nail, a rotational clamp device was devised using a rectangular opening to form a cross-rotation (Fig. 1E).

#### Assessment of the physical properties

The material tensile strength and fracture elongation were measured according to GB/T 1040.1-2006 using a universal material testing machine (Tinius Olsen, U.S.A.). The material bending strength and bending modulus were measured according to GB/T 9341-2008, and the material notch impact strength was measured according to GB/T 1843-2008. The material hardness was determined according to GB/T 2411-2008. The material thermal expansion coefficient was determined using a plastic polymer material expansion coefficient tester (GCPZY-100, China) according to GB/T 1036-89. GB/T standards, also known as Guo Biao/Tui Jian Biao, are the National Standards of the People's Republic of China. These standards, formulated and published by the Standardization Administration of China, encompass technical specifications, test methods, and product standards across various fields.

#### Isolation, cultivation, and identification of bone marrow mesenchymal stem cells

Bone marrow mesenchymal stem cells (BMSCs) were isolated from the bone marrow of both femurs and tibias of 5-week-old male rats. The isolated BMSCs were then cultured in a complete DMEM medium containing 10% fetal bovine serum (FBS, Gibco, U.S.A.) and 1% (v/v) penicillin/streptomycin and incubated at 37 °C and 5% CO_2_. After 24 h, the medium was replaced and non-adherent cells were discarded. The medium was changed every 3 days until the cell fusion rate reached 80%. Passage-3 BMSCs were used for all experiments. The identification of cell surface markers (CD29, CD90) of BMSCs was performed using flow cytometry (FACS Calibur, U.S.A.). The BMSCs were cultured in an osteogenic differentiation medium for 14 days and stained for alkaline phosphatase activity at 7 days and with Alizarin Red at 14 days to assess their osteogenic potential.

#### Cytotoxicity of compression nails

Cell toxicity was evaluated using the CCK-8 assay (TargetMol, U.S.A.). The sterilized femoral compression nails were immersed in α-minimum essential medium (α-MEM, Gibco, U.S.A.) complete medium for 72 h, and then the BMSCs cell suspension was evenly planted in a 96-well plate with a volume of approximately 100 μL per well. The plate was placed in a culture incubator for 24 h (37 °C, 5% CO_2_). One group was randomly selected and added with 100 μL/each well of α-MEM complete medium, while the other group was added with 100 μL/each well of plant material soaking liquid. The CCK-8 cell toxicity test was performed on 1, 3, 5 and 7 days after inoculation, and the data were recorded and the curve was plotted using an enzyme label instrument (450 nm, 37 °C, FLUO star Omega, Germany). Simultaneously, cell counts were performed alongside the CCK-8 assay, and the data were used to construct additional growth curves. Moreover, the total protein content of the cells was quantitatively determined using the bicinchoninic acid (BCA, CWBIO, China) assay and plotted on a curve.

#### Experimental animals and design

**Fig. 2:**
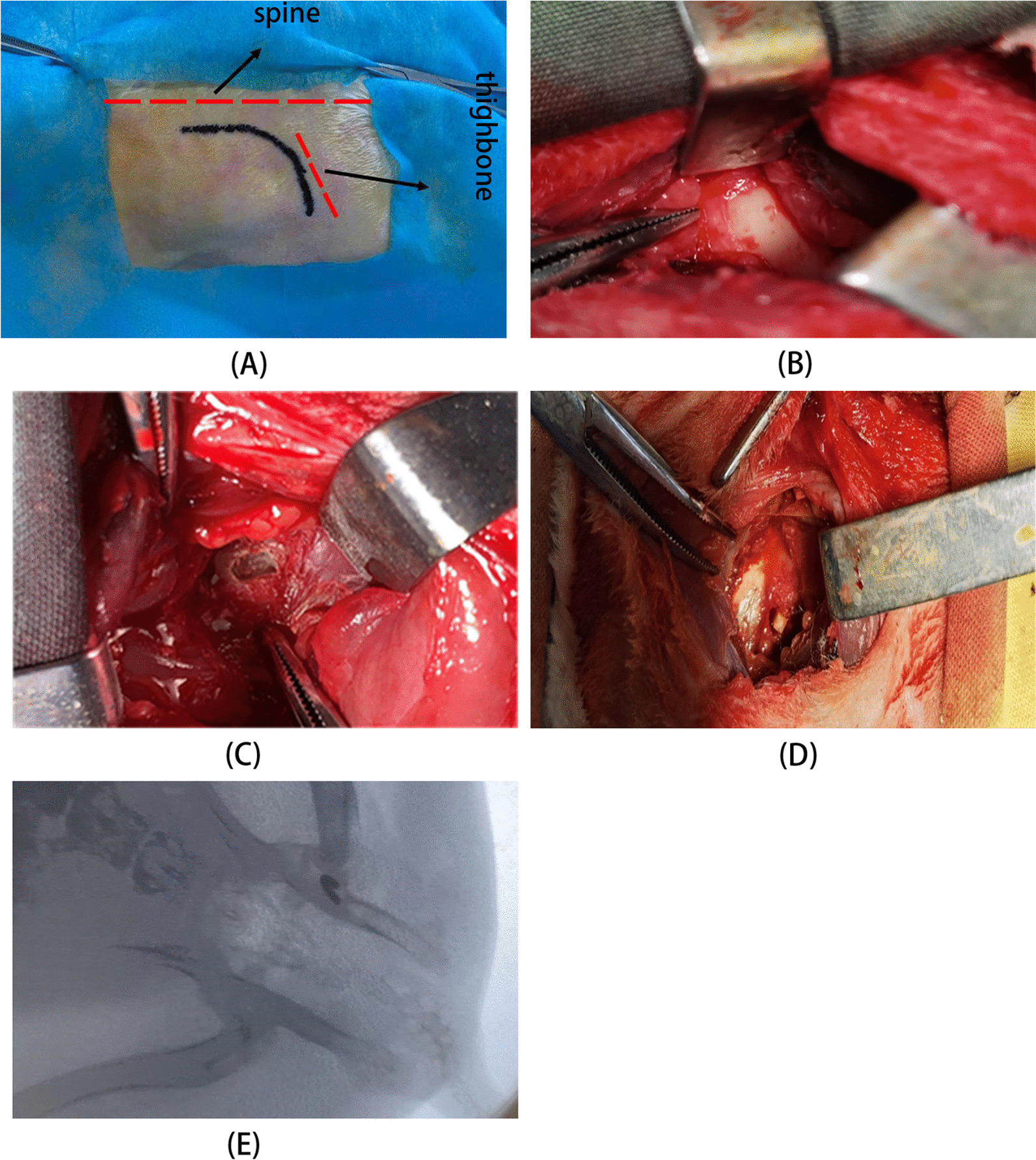
Operational procedures for creating the femoral head necrosis model. **A** Selection of the iliac crest and greater trochanter as the sites for an arched incision; **B** Sequential dissection of muscle and other soft tissues to expose the acetabulum; **C** Create a window approximately 3 mm away from the acetabular rim; **D** Implantation of the femoral head compression device. **E** Intraoperative X-ray imaging confirms the accurate placement of the bone window

The animal ethics of this experiment were approved by the Ethics Committee of Xi'an Jiaotong University School of Medicine (Ethics Approval No.: 2021–1413). Thirty-six female New Zealand white rabbits, weighing 4.0–5.0 kg and aged 12 months, were purchased from the Animal Experiment Center of Xi'an Jiaotong University and housed under standard conditions. The rabbits were randomly divided into six groups (A_1_–A_4_ and B_1_–B_2_ *n* = 6). Except for the B_2_ group which is the control group, all rabbits received a single ear vein injection of lipopolysaccharide (LPS) at a dose of 10 μg/kg at 0 h, followed by an intramuscular injection of methylprednisolone (MPS) at a dose of 20 mg/kg at 24 h, 48 h, and 72 h. After 3 weeks, 3D-printed compression devices with different curvatures (3.0 mm, 3.5 mm, 4.0 mm, and 4.5 mm) were implanted in groups A_1_-A_4_, group A_1_ was implanted with a 4.5 mm compression device, Group A_2_ with a 4.0 mm compression device, Group A_3_ with a 3.5 mm compression device, and Group A_4_ with a 3.0 mm compression device (Fig. 2A–D). Confirm the placement of the compression device intraoperatively using an X-ray (Fig. 2E), with a local injection of 0.2% ropivacaine for analgesia. Group B animals underwent only an incision without implantation of the compressive device and received an intramuscular injection of penicillin at a dose of 400,000 U per day for 3 days after surgery. At 8 weeks post-surgery, the modeling effect was evaluated using micro-computed tomography (micro-CT, Siemens Healthineers, Germany). After euthanasia, the femoral head specimens were subjected to micro-CT scanning and reconstruction following decalcification, and pathological sections were prepared.

#### Gross observation of the specimens

After 8 weeks, femoral head specimens were harvested to observe the contour, color, shape, and smoothness of the femoral head in each group.

#### Micro-computed tomography (micro-CT) imaging system was used for evaluation

Femoral heads were subjected to fixation in paraformaldehyde for 48 h before imaging using a micro-CT system, which was configured with scanning parameters including a voltage of 70 V, a current of 100 mA, and a pixel size of 10 μm. Subsequently, the acquired images were analyzed and processed using VG Studio MAX software. Three-dimensional reconstruction techniques available in VG Studio MAX were utilized to quantitatively assess various parameters within the subchondral bone region of the femoral head. These parameters included the ratio of bone volume (BV) to total volume (TV), trabecular number, and trabecular thickness.

### Histological analysis

Cultured specimens were subjected to fixation in 4% paraformaldehyde for 24 h at 4 °C, followed by decalcification in a 20% EDTA solution. Subsequently, the specimens were dehydrated and embedded in paraffin, and 10-μm-thick sections were prepared. The sections were then stained with hematoxylin and eosin (HE) for observation under an optical microscope, and the number of empty lacunae in the subchondral bone region of the femoral head was quantified.

### Statistical analysis

All experimental data analysis was performed using independent-sample t-tests, with statistical analysis conducted using SPSS 24.0 software. A significance level of *P* < 0.05 was considered statistically significant.

## Results

### Characterization of the physical properties

The femoral head compression device has an opaque white appearance and was constructed using a 3D printing technique with a layer thickness of 0.1 mm and a density of 1.13 g/cm^3^. Physical characterization of the device material was performed, and the results are summarized in Table 1.

**Table 1:** The physical properties of the femoral compression screw

Project	Method	Numeric value
Tensile strength	GB/T 1040.1-2006	51 MPa
Elongation at break	GB/T 1040.1-2006	21%
Flexural strength	GB/T 9341-2008	65–80 MPa
Flexural modulus	GB/T 9341-2008	1800–2200 MPa
Impact strength notched Izod	GB/T 1843-2008	100 J/m
Shore hardness	GB/T 2411-2008	81 D
Coefficient of thermal expansion	GB/T 1036-89	60 μm/m °C

### Identification of bone marrow mesenchymal stem cells

**Fig. 3:**
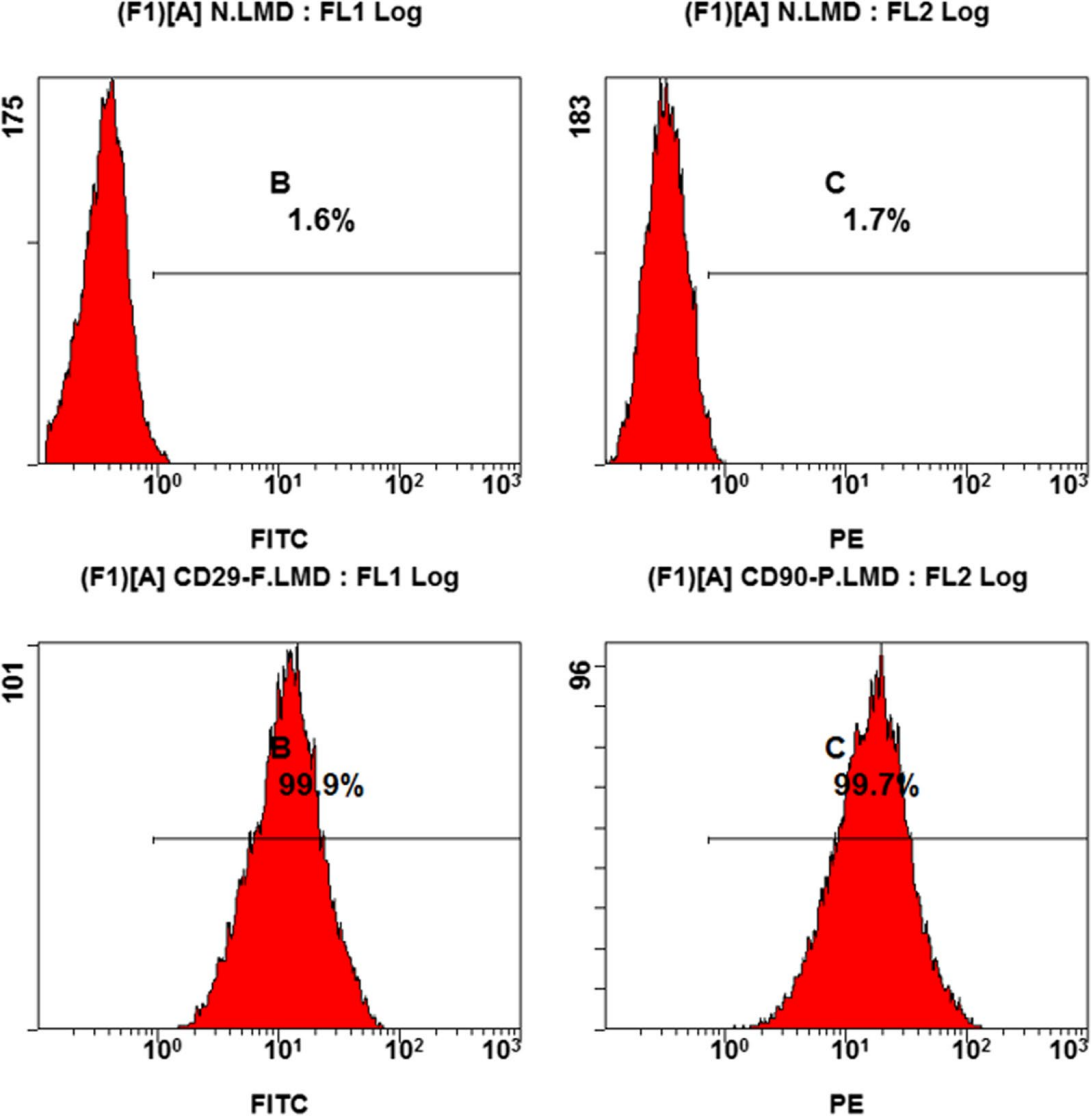
Flow cytometry analysis was performed using surface markers CD29 and CD90 to characterize the cells

To identify the isolated cells, the characterization of the cells using flow cytometry revealed positive expression rates of 99.9% and 99.7% for CD29 and CD90, respectively, with negative expression rates of 1.6% and 1.7% (Fig. 3).

**Fig. 4:**
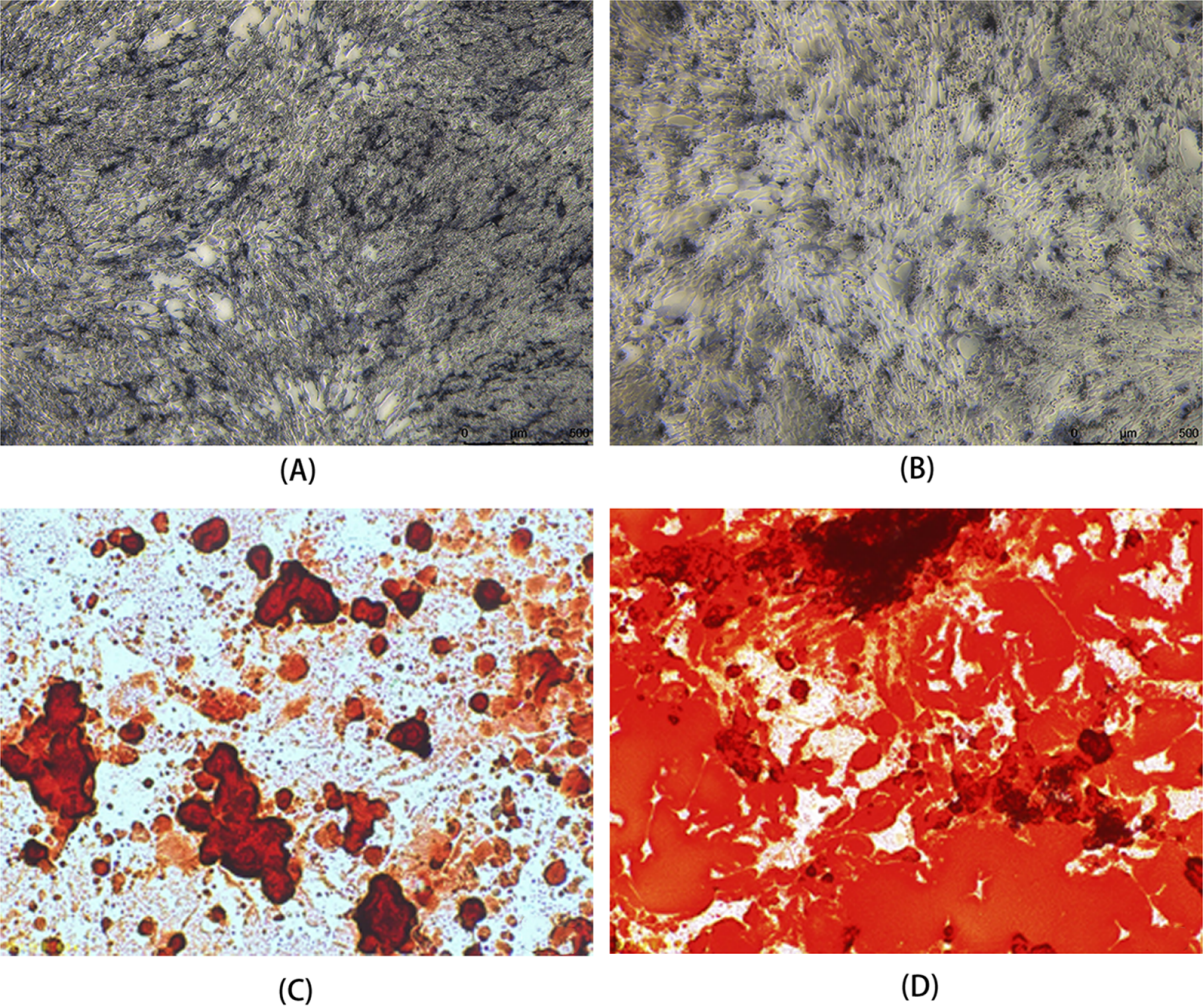
ALP staining status after 7 days of osteogenic induction and the safranin-O staining status after 14 days of osteogenic induction. **A** Untreated BMSCs exhibited lower alkaline phosphatase activity as demonstrated by ALP staining. **B**) BMSCs subjected to osteogenic induction displayed higher alkaline phosphatase activity, as indicated by ALP staining. **C** Safranin-O staining of untreated BMSCs revealed less calcium deposition. **D** osteogenically induced BMSCs displayed substantial calcium deposition

Upon culturing the aforementioned isolated cells in the osteogenic induction medium (OIM) for a duration of 7 days, cells were qualitatively observed to exhibit greater ALP staining compared to their non-induced counterparts. The OIM solution was prepared by supplementing the previously prepared complete DMEM basal medium with 1% glutamine, 0.2% ascorbic acid, 1% β-glycerophosphate, and 0.01% dexamethasone. Cytoplasmic granules and precipitates were observed in abundance and appeared deeply stained (Fig. 4A, B). These results indicate the potential for osteogenic differentiation of the isolated BMSCs.

The ARS staining solution was utilized for the detection of osteoblastic differentiation, as it can bind with calcium deposits on the surface of osteoblasts that have undergone successful osteogenic induction. Following 14 days of osteogenic induction in vitro, the isolated and cultured cells were subjected to ARS staining. The results of ARS staining revealed significantly larger mineralized nodules in osteogenically induced BMSCs, as compared to non-induced BMSCs (Fig. 4C, D). Taken together, these findings suggest that the isolated BMSCs possess the capability to differentiate into osteoblasts upon osteogenic induction.

### Assessment of cytotoxicity of the femoral head compression device

**Fig. 5:**
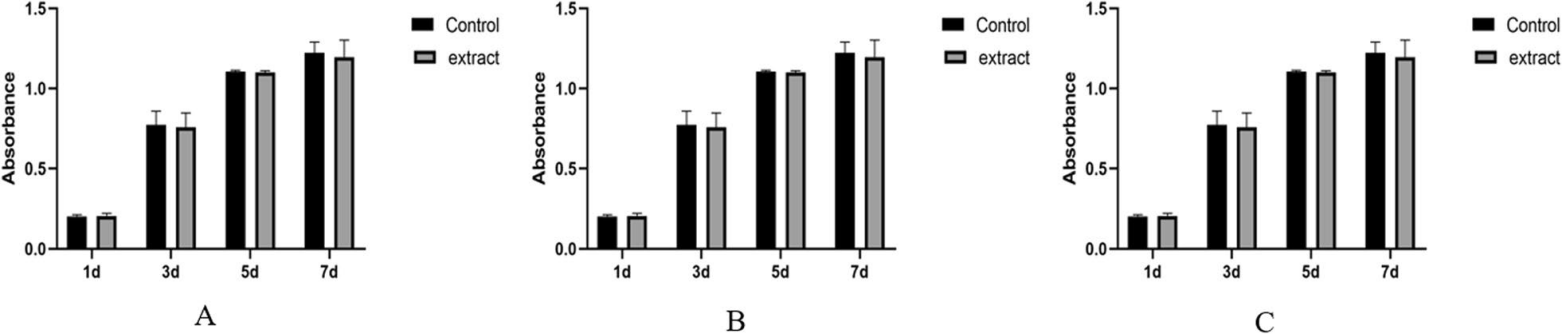
Device toxicity assessment. **A** The results of the CCK-8 assay revealed no significant statistical difference in cell viability between the two groups at days 1, 3, 5, and 7 of cell culture. **B** The cell counting results demonstrated no significant statistical difference in cell numbers between the two groups at days 1, 3, 5, and 7 of cell culture. **C** The BCA assay results indicated no significant statistical difference in total protein content between the two groups at days 1, 3, 5, and 7 of cell culture

Cellular toxicity analysis of the immersion solution of the device was performed using the CCK-8 assay, cell counting method, and BCA assay. The results demonstrated that the cell viability of cells cultured in the immersion solution of the pressure device remained unaffected on days 1, 3, 5, and 7 of cell culture (Fig. 5A). Additionally, no significant statistical difference in cell count was observed between the two groups (Fig. 5B). Moreover, the quantitative analysis of total cellular protein showed no significant statistical difference between the two groups (Fig. 5C). Therefore, these findings collectively indicate that the materials of the device are non-cytotoxic.

### Macroscopic examination

**Fig. 6:**
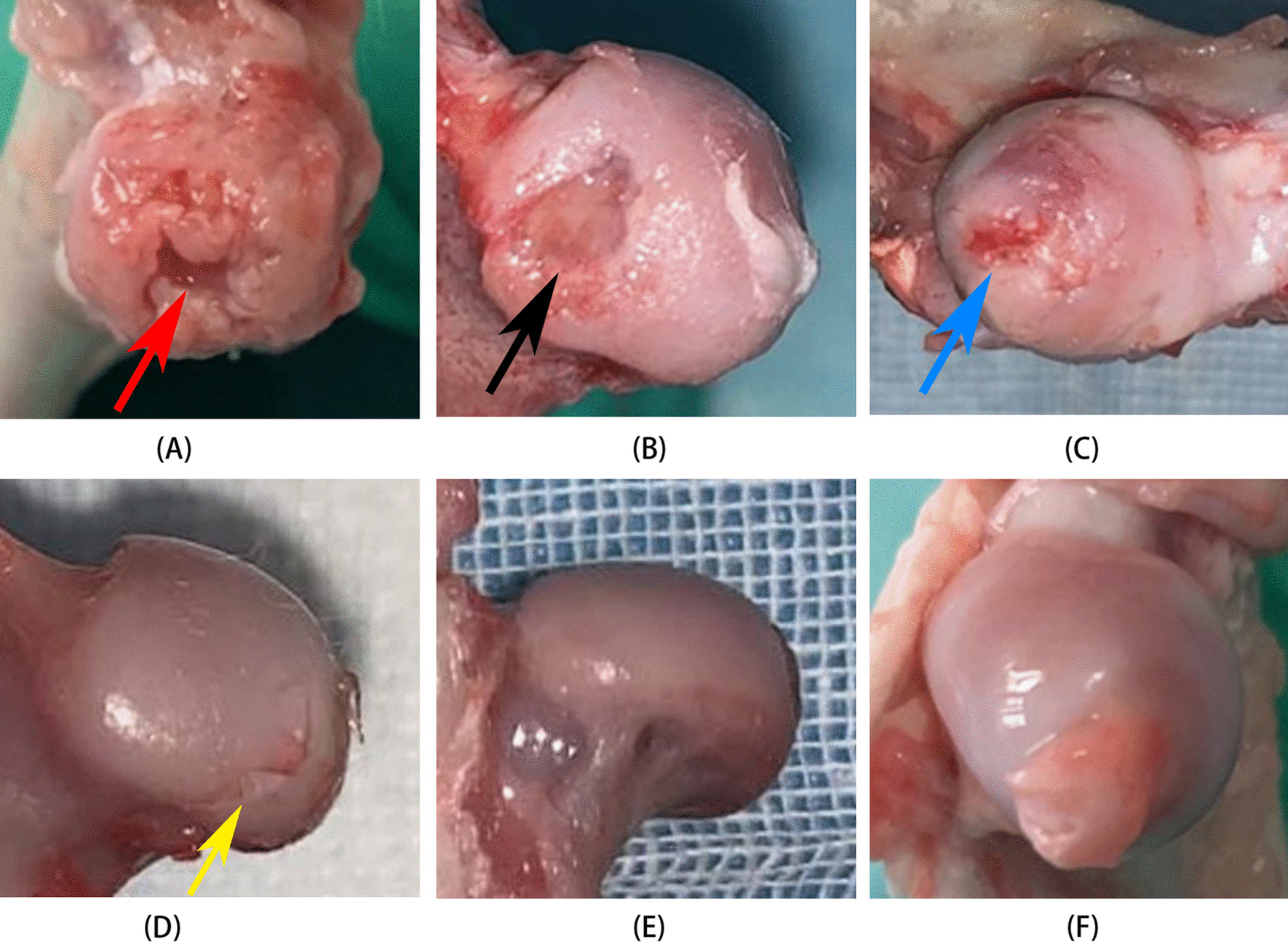
External morphology of the femoral heads in each group. **A** The femoral head in the A1 group: complete collapse with accompanying bone defect (red arrow); **B** the femoral head in the A2 group: significant cartilage loss and minor femoral head collapse (black arrow); **C** the femoral head in the A3 group: localized cartilage defect (blue arrow); **D** the femoral head in the A4 group: minor cartilage delamination (yellow arrow); **E** the femoral head in the B1 group; **F** the femoral head in the B2 group

After 8 weeks, the femoral head specimens were obtained and morphological changes were observed in each group. The femoral head of the control group B_2_ had a normal appearance with a smooth and intact cartilage surface, no collapse, and a white color (Fig. 6F). The femoral head of group B_1_ had a normal contour, relatively smooth cartilage without defects, and a darker color compared with the control group (Fig. 6E). The femoral head of group A_4_ was darker in color (Fig. 6D) than that of the control group, with small areas of cartilage peeling off (yellow arrow). In group A_3_, a focal cartilage defect (blue arrow) was observed in the compression screw site of the femoral head (Fig. 6C). Although the contour of the femoral head in group A_2_ was normal (Fig. 6B), large areas of cartilage defects and slight collapse of the femoral head (black arrow) were observed in the compression screw site. The femoral head contour was completely lost in group A_1_ (Fig. 6A), and severe femoral head collapse and cartilage damage were observed (red arrow).

### Results of micro-CT analysis

**Fig. 7:**
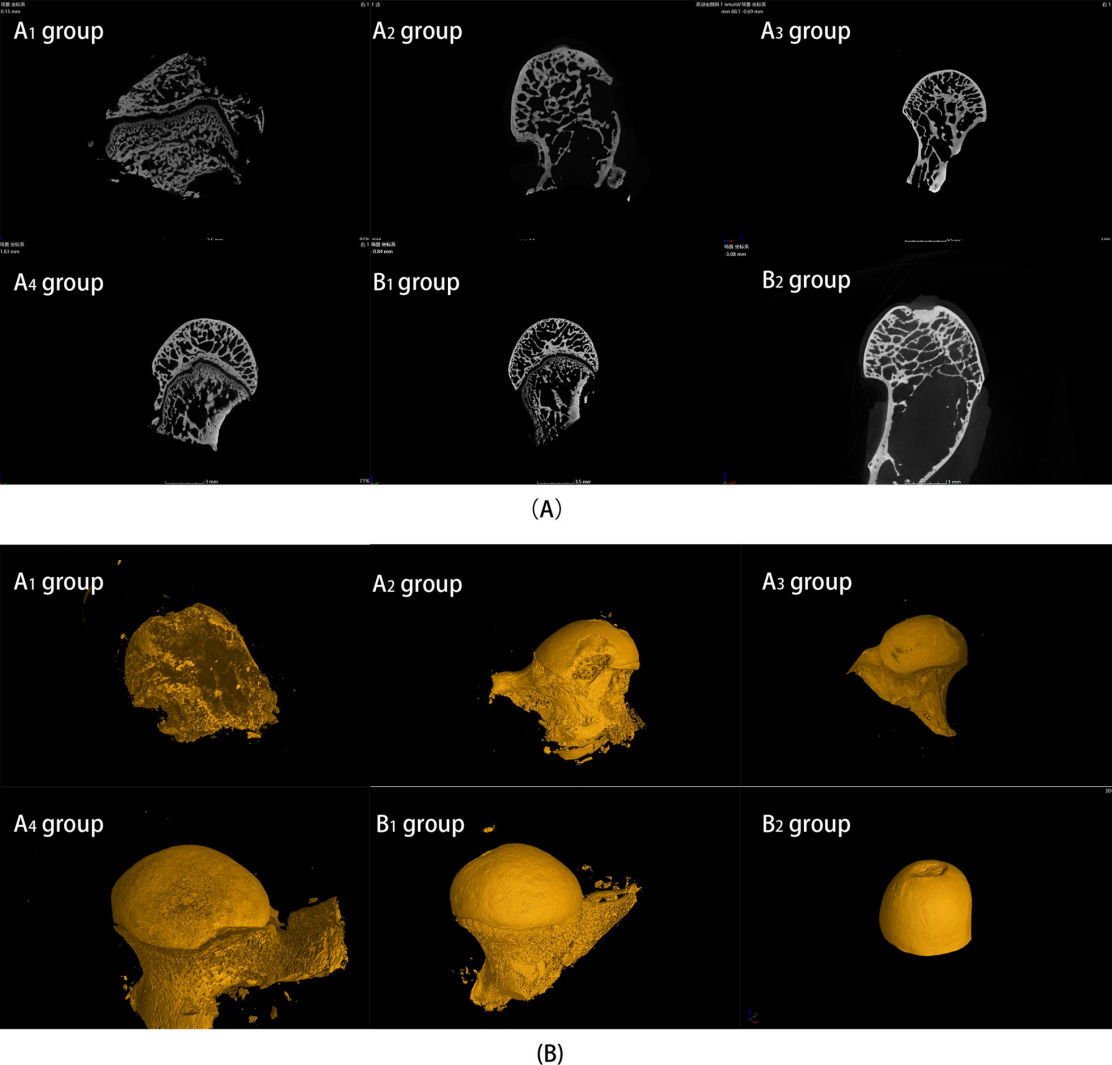
The micro-CT images depict the modeling effects of the femoral head compression device implanted in hip sockets with varying radii of curvature. **A** Coronal micro-CT scan results in the image, **B** micro-CT 3D reconstruction image

After 8 weeks of modeling, the rabbits in each group were euthanized, and the femoral heads on the operated side were harvested and subjected to micro-CT scanning. The micro-CT results (Fig. 7A) revealed a notable reduction in trabecular bone volume and density in the subchondral bone of all modeled groups compared to the blank control group B_2_. Furthermore, there was a marked decrease in the thickness and density of the trabecular bone, as well as an increase in spacing. Specifically, the femoral head collapse was observed in A_2_, and severe femoral head collapse with the bone defect was observed in A_1_, while no significant collapse was observed in the femoral heads of B_1_, B_2_, A_3_, and A_4_ groups. Subsequent analysis of micro-CT data using software (Fig. 7B) confirmed that all modeled groups exhibited a significant decrease in the bone volume to total volume ratio, a reduction in the number of trabecular bones, and an increase in the trabecular spacing when compared to the control group B_2_.

### Histological analysis

**Fig. 8:**
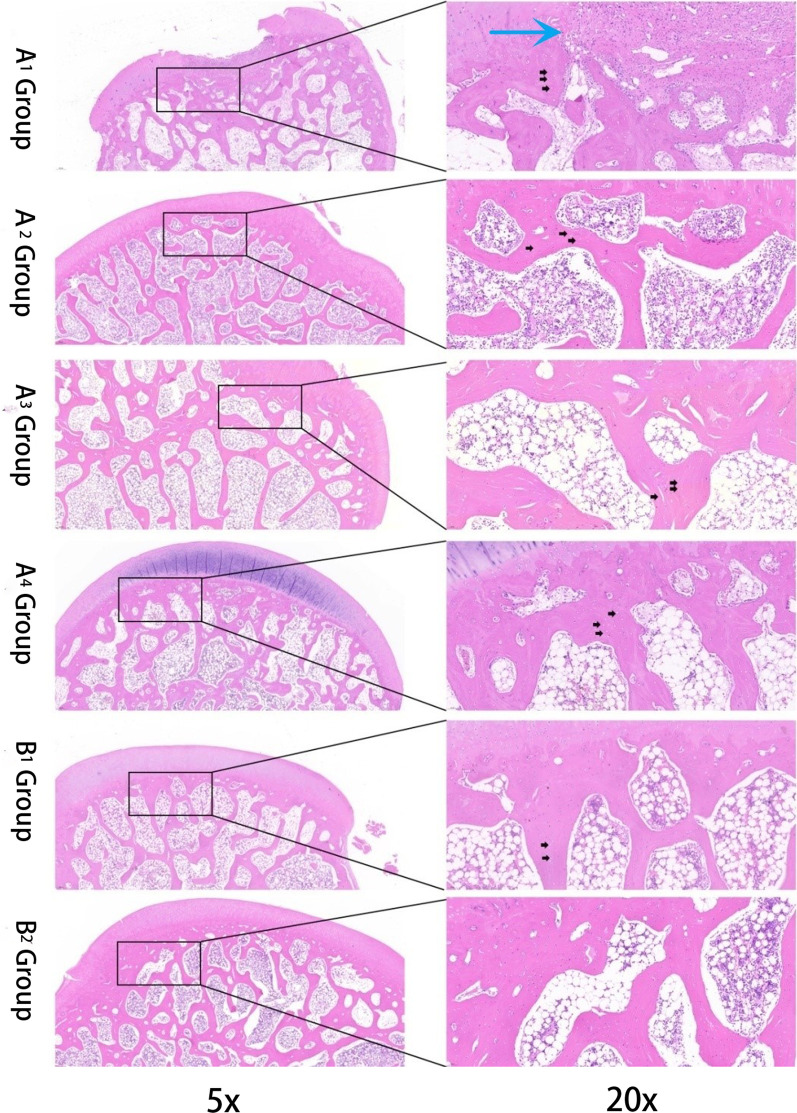
The findings obtained from HE staining: osteolytic lesions (black arrow); infiltration of inflammatory cells (blue arrow)

After euthanizing New Zealand white rabbits at 8 weeks post-surgery, the femoral heads on the operated side were decalcified, sectioned, and subsequently subjected to HE staining. The histological evaluation of the HE-stained sections (Fig. 8) revealed a significant loss of subchondral bone in all experimental groups as compared to the control group, as evidenced by the presence of numerous osteolytic lesions (black arrows). Notably, the area of femoral head collapse in the A_1_ group showed marked infiltration of inflammatory cells (blue arrow). These histological findings corroborate the micro-CT results, revealing a correlation between the degree of femoral head necrosis in groups A1–A4 and the curvature radius of the loading device. Specifically, we observed that a larger curvature radius was associated with a higher degree of femoral head necrosis, further supporting the existing correlation between these factors.

## Discussion

Over the past century, the pathogenesis of ONFH has evolved from the initial speculation that it was associated with vascular injury to later theories involving fat embolism and other mechanisms. Currently, research has advanced to the genetic level, and investigations have never ceased [17]. Research tools for ONFH have evolved from gross specimens to include various animal models such as mice [18], rabbits [13], pigs [19], and ostriches [20], among others, which have contributed to a diversity of research methods. However, a review of the current research reveals that there are still some shortcomings in the study of ONFH. For instance, as mentioned in Introduction, there are significant differences between the hip joint-femoral head structure of quadrupeds and that of humans, and their load characteristics differ considerably. Therefore, it is difficult to simulate the real state of femoral head load-bearing from a biomechanical perspective [21]. Moreover, bipeds are mostly non-mammals, which have significant differences in hormone sensitivity compared to mammals, making it difficult to simulate the natural course of ONFH induced by hormones [12]. Animal models are the foundation of basic medical research, and therefore, based on the needs of the research, it is desirable to improve the existing models through some technical means, to open up new directions for the study of ONFH.

We selected rabbits as the animal model for a novel ONFH model primarily due to their unique anatomical characteristics. Specifically, the rabbit hip socket is positioned near the surface on both sides of the spine, allowing for the posterior entry and exposure of the lateral aspect of the hip socket without the need for a muscle incision. This technique reduces surgical trauma and subsequent errors during modeling. Additionally, the larger femoral head size of rabbits, coupled with stronger femoral ligaments and joint capsules, facilitates consistent positive pressure interaction with the compression device. Notably, this force remains unaffected by positional changes [22], enabling an accurate simulation of femoral head stress in the upright human posture and enhancing the femoral head necrosis model.

In the extant literature, a plethora of techniques have been utilized to induce ONFH, such as vascular occlusion [13, 23], steroid administration [24–26], cryogenic insult [27], dislocation of the hip joint [28], exposure to high pressure [29], hypersensitivity reactions [30], endotoxin injection [31], and various combinations thereof. However, the majority of animal models fail to develop advanced-stage femoral head collapse and instead perish as a result of invasive systemic injuries leading to sepsis or death [32]. Therefore, the judicious production of localized subchondral necrosis within the femoral head has emerged as a pivotal strategy in the establishment of a robust ONFH model. To this end, the fabrication of internal bone devices represents a promising avenue of investigation in this field. Reed et al. developed a cryoprobe that was guided by radiography to induce necrosis in the subchondral bone [33]. Goetz further validated the effectiveness of this technique by conducting a finite element analysis on the necrotic area [34, 35]. Another study by Fan used a more advanced cryo-heating probe to create an ONFH model [36]. However, due to the size and cost of this equipment, the use of this technique is limited in establishing ONFH models.

In the present study, we developed a pressurized device with dimensions comparable to the hip joint gap to apply a compressive load to the femur. Additionally, a rotating clamping device was designed to apply the load to the upper lateral aspect of the femoral head. The pressurized device was fabricated using 3D printing technology with resin as the material. The experimental findings indicate that the resin material employed for modeling does not exhibit any cellular toxicity. Femoral head specimens modeled with steroids (A_1_-A_4_, B_1_) exhibit a considerable number of empty osteocyte lacunae on HE-stained sections compared to the control group (B_2_). The application of the pressurized device (A_1_-A_4_) results in localized cartilage defects and subchondral bone fractures in the pressurized device region, leading to femoral head collapse. Additionally, the extent of femoral head damage varies with the curvature radius of the pressurized device, which can be modified to simulate different stages of femoral head necrosis. For instance, in the early stages of femoral head osteonecrosis, human pathological observations have revealed subtle morphological alterations accompanied by localized cartilage detachment. This corresponds to the femoral head damage state observed in Group A4 of our study. During the intermediate phase of femoral head osteonecrosis, which represents the period when a majority of patients seek medical intervention, human pathological investigations have demonstrated mild to moderate collapse of the femoral head while preserving the joint space. This parallels the femoral head damage state observed in Groups A3 and A4 of our study. In the advanced stage of femoral head osteonecrosis, human pathological examinations have documented significant femoral head collapse, mirroring the femoral head damage state observed in Group A1 of our study.

Nonetheless, this experiment is subject to certain limitations. Firstly, the study solely focused on a single observation point at 8 weeks post-surgery, which precluded the continuous monitoring of the progression of femoral head necrosis. Secondly, the procedure for inserting the pressurized device necessitated adequate preoperative training.

## Conclusion

This study has developed a novel method for modeling femoral head necrosis that incorporates stress factors using 3D printing technology and principles of biomechanics. This approach enhances the fidelity of the model to the clinical scenario and improves its utility as an experimental platform for osteonecrosis of the femoral head. The technique holds promise for advancing our understanding of the pathogenesis and potential therapeutic interventions for this debilitating condition.

## Data Availability

All data generated or analyzed during this study are included in this published article.

## References

[1] Zhao D, Zhang F, Wang B, Liu B, Li L, Kim SY (2020). Guidelines for clinical diagnosis and treatment of osteonecrosis of the femoral head in adults (2019 version). J Orthop Translat.

[2] Sadile F, Bernasconi A, Russo S, Maffulli N (2016). Core decompression versus other joint preserving treatments for osteonecrosis of the femoral head: a meta-analysis. Br Med Bull.

[3] Quaranta M, Miranda L, Oliva F, Aletto C, Maffulli N (2021). Osteotomies for avascular necrosis of the femoral head. Br Med Bull.

[4] Hines JT, Jo WL, Cui Q, Mont MA, Koo KH, Cheng EY (2021). Osteonecrosis of the femoral head: an updated review of ARCO on pathogenesis, staging and treatment. J Korean Med Sci.

[5] Cui Q, Jo WL, Koo KH, Cheng EY, Drescher W, Goodman SB (2021). ARCO consensus on the pathogenesis of non-traumatic osteonecrosis of the femoral head. J Korean Med Sci.

[6] Migliorini F, La Padula G, Oliva F, Torsiello E, Hildebrand F, Maffulli N (2022). Operative management of avascular necrosis of the femoral head in skeletally immature patients: a systematic review. Life (Basel)..

[7] Migliorini F, Maffulli N, Eschweiler J, Tingart M, Baroncini A (2021). Core decompression isolated or combined with bone marrow-derived cell therapies for femoral head osteonecrosis. Expert Opin Biol Ther.

[8] Hua KC, Yang XG, Feng JT, Wang F, Yang L, Zhang H (2019). The efficacy and safety of core decompression for the treatment of femoral head necrosis: a systematic review and meta-analysis. J Orthop Surg Res.

[9] Migliorini F, Maffulli N, Baroncini A, Eschweiler J, Tingart M, Betsch M (2023). Prognostic factors in the management of osteonecrosis of the femoral head: a systematic review. Surgeon.

[10] Li Y, Han R, Geng C, Wang Y, Wei L (2009). A new osteonecrosis animal model of the femoral head induced by microwave heating and repaired with tissue engineered bone. Int Orthop.

[11] Zhu ZH, Gao YS, Luo SH, Zeng BF, Zhang CQ (2011). An animal model of femoral head osteonecrosis induced by a single injection of absolute alcohol: an experimental study. Med Sci Monit..

[12] Xu J, Gong H, Lu S, Deasey MJ, Cui Q (2018). Animal models of steroid-induced osteonecrosis of the femoral head—a comprehensive research review up to 2018. Int Orthop.

[13] Tudisco C, Botti F, Bisicchia S, Ippolito E (2015). Ischemic necrosis of the femoral head: an experimental rabbit model. J Orthop Res.

[14] Jiang W, Wang P, Wan Y, Xin D, Fan M (2015). A simple method for establishing an ostrich model of femoral head osteonecrosis and collapse. J Orthop Surg Res.

[15] Floerkemeier T, Lutz A, Nackenhorst U, Thorey F, Waizy H, Windhagen H (2011). Core decompression and osteonecrosis intervention rod in osteonecrosis of the femoral head: clinical outcome and finite element analysis. Int Orthop.

[16] Ma JX, He WW, Zhao J, Kuang MJ, Bai HH, Sun L (2017). Bone microarchitecture and biomechanics of the necrotic femoral head. Sci Rep.

[17] Mont MA, Cherian JJ, Sierra RJ, Jones LC, Lieberman JR (2015). Nontraumatic osteonecrosis of the femoral head: Where do we stand today? A ten-year update. J Bone Joint Surg Am.

[18] Weinstein RS, Nicholas RW, Manolagas SC (2000). Apoptosis of osteocytes in glucocorticoid-induced osteonecrosis of the hip. J Clin Endocrinol Metab.

[19] Zhang P, Liang Y, Kim H, Yokota H (2010). Evaluation of a pig femoral head osteonecrosis model. J Orthop Surg Res.

[20] Qin L, Yao D, Zheng L, Liu WC, Liu Z, Lei M (2015). Phytomolecule icaritin incorporated PLGA/TCP scaffold for steroid-associated osteonecrosis: proof-of-concept for prevention of hip joint collapse in bipedal emus and mechanistic study in quadrupedal rabbits. Biomaterials.

[21] Li Z, Shao W, Lv X, Wang B, Han L, Gong S (2023). Advances in experimental models of osteonecrosis of the femoral head. J Orthop Transl.

[22] Greenaway JB, Partlow GD, Gonsholt NL, Fisher KR (2001). Anatomy of the lumbosacral spinal cord in rabbits. J Am Anim Hosp Assoc.

[23] Norman D, Reis D, Zinman C, Misselevich I, Boss JH (1998). Vascular deprivation-induced necrosis of the femoral head of the rat. An experimental model of avascular osteonecrosis in the skeletally immature individual or Legg–Perthes disease. Int J Exp Pathol..

[24] Drescher W, Weigert KP, Bünger MH, Ingerslev J, Bünger C, Hansen ES (2004). Femoral head blood flow reduction and hypercoagulability under 24 h megadose steroid treatment in pigs. J Orthop Res.

[25] Cui Q, Wang GJ, Su CC, Balian G (1997). The Otto Aufranc Award. Lovastatin prevents steroid induced adipogenesis and osteonecrosis. Clin Orthop Relat Res..

[26] Yamamoto T, Irisa T, Sugioka Y, Sueishi K (1997). Effects of pulse methylprednisolone on bone and marrow tissues: corticosteroid-induced osteonecrosis in rabbits. Arthritis Rheum.

[27] Malizos KN, Quarles LD, Seaber AV, Rizk WS, Urbaniak JR (1993). An experimental canine model of osteonecrosis: characterization of the repair process. J Orthop Res.

[28] Nishino M, Matsumoto T, Nakamura T, Tomita K (1997). Pathological and hemodynamic study in a new model of femoral head necrosis following traumatic dislocation. Arch Orthop Trauma Surg.

[29] Lehner CE, Adams WM, Dubielzig RR, Palta M, Lanphier EH (1997). Dysbaric osteonecrosis in divers and caisson workers. An animal model. Clin Orthop Relat Res..

[30] Newton B, Crawford CJ, Powers DL, Allen BL (1994). The immature goat as an animal model for Legg–Calvé–Perthes disease. J Investig Surg.

[31] Kawamoto S, Shirai N, Strandberg JD, Boxerman JL, Bluemke DA (2000). Nontraumatic osteonecrosis: MR perfusion imaging evaluation in an experimental model. Acad Radiol.

[32] Irisa T, Yamamoto T, Miyanishi K, Yamashita A, Iwamoto Y, Sugioka Y (2001). Osteonecrosis induced by a single administration of low-dose lipopolysaccharide in rabbits. Bone.

[33] Reed KL, Brown TD, Conzemius MG (2003). Focal cryogen insults for inducing segmental osteonecrosis: computational and experimental assessments of thermal fields. J Biomech.

[34] Goetz JE, Pedersen DR, Robinson DA, Conzemius MG, Baer TE, Brown TD (2008). The apparent critical isotherm for cryoinsult-induced osteonecrotic lesions in emu femoral heads. J Biomech.

[35] Goetz JE, Robinson DA, Pedersen DR, Conzemius MG, Brown TD (2011). Cryoinsult parameter effects on the histologically apparent volume of experimentally induced osteonecrotic lesions. J Orthop Res.

[36] Fan M, Peng J, Wang A, Zhang L, Liu B, Ren Z (2011). Emu model of full-range femoral head osteonecrosis induced focally by an alternating freezing and heating insult. J Int Med Res.

